# Single-port laparoscopic colectomy versus conventional laparoscopic colectomy for colon cancer: a comparison of surgical results

**DOI:** 10.1186/1477-7819-10-61

**Published:** 2012-04-24

**Authors:** Hiroyuki Egi, Minoru Hattori, Takao Hinoi, Yuji Takakura, Yasuo Kawaguchi, Manabu Shimomura, Masakazu Tokunaga, Tomohiro Adachi, Takashi Urushihara, Toshiyuki Itamoto, Hideki Ohdan

**Affiliations:** 1 Department of Surgery, Division of Frontier Medical Science, Programs for Biomedical Research, Graduate School of Biomedical Sciences, Hiroshima University, 1-2-3 Kasumi, Minami-ku, Hiroshima 734-8551, Japan; 2 Advanced Medical Skills Training Center, Graduate School of Biomedical Sciences, Hiroshima University, 1-2-3 Kasumi, Minami-ku, Hiroshima 734-8551, Hiroshima, Japan; 3 Department of Surgery, Hiroshima Prefectural Hospital, 1-5-54 Ujina-Kanda, Minami-ku, Hiroshima 734-8530, Japan

**Keywords:** Single-port laparoscopic surgery, Single-incision laparoscopic surgery, Conventional laparoscopic surgery, Laparoscopic colectomy, Colon cancer, Gelport

## Abstract

**Background:**

Single-port laparoscopic surgery is a new technique that leaves no visible scar. This new technique has generated strong interest among surgeons worldwide. However, single-port laparoscopic colon surgery has not yet been standardized. Our aim in this study was to evaluate the feasibility of single-port laparoscopic colectomy compared with conventional laparoscopic colectomy for colon cancer.

**Methods:**

We conducted a case-matched, controlled study comparing single-port laparoscopic colectomy to conventional laparoscopic colectomy for right-sided colon cancer.

**Results:**

A total of ten patients were included for the single-port laparoscopic colectomy (S-LAC) group and ten patients for the conventional laparoscopic colectomy (C-LAC) group. The length of the skin incision in the S-LAC group was significantly shorter than that of the C-LAC group.

**Conclusion:**

Our early experiences indicated that S-LAC for right-sided colon cancer is a feasible and safe procedure and that S-LAC results in a better cosmetic outcome.

## Background

Laparoscopic surgery has been a standard strategy for a variety of gastrointestinal diseases. The first report about laparoscopic colectomy was published by Jacobs *et al. *[1] two decades ago. Since then the use of laparoscopic colectomy for colon cancer has gradually increased, and it is now acceptable treatment not only for early colon cancer but also for advanced cases because of its oncological safety and feasibility [2,3]. Recently, natural orifice transluminal endoscopic surgery (NOTES) has been studied as the next generation of minimally invasive surgery. This new technique was described for the first time by Kalloo *et al*., who introduced their work performing transgastric peritoneoscopy in a porcine model [4]. Marescaux *et al. *also reported successful NOTES in a clinical case [5]. However, the feasibility and safety of NOTES have not been evaluated. Single-port laparoscopic surgery is also a new technique which leaves no visible scar. This new technique has generated interest among surgeons worldwide. Although the use of single-port laparoscopic cholecystectomy has spread rapidly, single-incision laparoscopic colon surgery has not yet been standardized. Our aim in this study was to evaluate the feasibility of single-port laparoscopic colectomy compared with conventional laparoscopic colectomy for colon cancer which requires D2 lymph node dissection.

## Methods

This study was performed with permission of the Ethics Committee of the Hiroshima University.

We conducted a case-matched, controlled study comparing single-port laparoscopic colectomy to conventional laparoscopic colectomy for right-sided colon cancer. The inclusion criteria were right-sided colon cancer which required colon resection with D2 lymph node dissection. The single-port laparoscopic colectomy group included selected patients who completed their treatment between February 2010 and March 2011 (*n *= 10). Patients who underwent conventional laparoscopic surgery for right-sided colon cancer between April 2006 and March 2010 were selected as the control group for this study (*n *= 10). These patients were matched with regard to the patient's age, sex, body mass index (BMI), American Society of Anesthesiologists (ASA) score, history of abdominal surgery, disease type and tumor location. No consideration or analysis of surgical parameters and outcomes was made until these groups were definitively selected as the best comparison cohort based only on preoperative variables.

### Surgical technique

**Figure 1:**
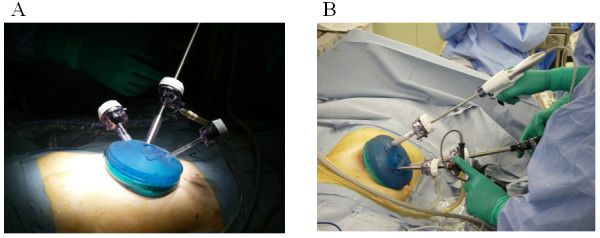
**The Gelport was used as the access port**. The flexible videolaparoscope, the Harmonic Ace energy source and other laparoscopic instruments used were the same as those used in the conventional laparoscopic colectomy group.

After obtaining informed consent, we placed patients with right-sided colon cancer in the supine position. The surgical methods for both single-port laparoscopic colectomy (S-LAC) and conventional laparoscopic colectomy (C-LAC) were performed using a mediolateral approach, and the hand-sewn anastomoses were performed extracorporeally. In the S-LAC group, a 3-cm skin incision was made in the umbilicus and laparotomy was performed. The Gelport (Applied Medical, Rancho Santa Margarita, CA, USA) was inserted through this incision and used as the access port. We usually used three trocars of different sizes (Ethicon, Inc, Cincinnati, OH, USA) to prevent clashes between these trocars. The camera was a flexible videolaparoscope (Olympus Medical Systems Corp, Tokyo, Japan), and the energy source was the Harmonic Ace (Ethicon, Inc). The other laparoscopic instruments were the same as those used in conventional laparoscopic colonic surgery (Figure 1). For the C-LAC group, the first trocar was inserted through the infraumbilical incision, and another four trocars were inserted sequentially. After intracorporeal completion of the procedure, a small skin incision was made in the lower abdomen or umbilicus. All instruments used, including the camera and energy device, were the same in both the C-LAC and S-LAC groups.

The perioperative outcomes, including the surgical method, length of skin incision, length of operation, estimated blood loss and conversion rate to conventional laparoscopic surgery or open surgery, as well as the complications, were analyzed (Table 1). The pathological findings included the degree of differentiation, depth, presence of lymph node metastasis, lymphatic vessel invasion, vascular invasion and the number of lymph nodes resected, and these results were analyzed as well (Table 2).

**Table 1:** Perioperative outcomes^a^

	Laparoscopic colectomy for colon cancer		
**Parameters**	**S-LAC (*N *= 10)**	**C-LAC (*N *= 10)**	***P *value**

Method			0.141

Ileocecal resection	8	5	

Right hemicolectomy	1	5	

Transverse colectomy	1	0	

Operative time (minutes)	192.0 (156 to 231)	222.0 (44 to 244)	0.063

Estimated blood loss (ml)	48.0 (0 to 110)	51.5 (21 to 244)	0.190

Length of skin incision (cm)	3 (2 to 3)	5 (3 to 6)	< 0.001

Conversion rate (%)	0 (0%)	1 (10.0%)	0.474

Hospital stay after operation (days)	8.0 (6 to 13)	10.5 (7 to 21)	0.023

^a^C-LAC = conventional laparoscopic colectomy; S-LAC = single-port laparoscopic colectomy. Data are expressed as median (range) or as raw numbers.

**Table 2:** Pathological outcomes^a^

	Laparoscopic colectomy for colon cancer		
**Parameters**	**S-LAC (*N *= 10)**	**C-LAC (*N *= 10)**	***P *value**

Differentiation			0.661

Well	7	6	

Moderate	1	2	

Pap	1	0	

Well-differentiated endocrine carcinoma	0	1	

Adenoma	1	1	

Depth			0.459

m (membrane)	4	3	

sm (lymphatic invasion)	6	4	

mp (vascular invasion)	0	1	

a	0	2	

n			1.000

Negative	10	9	

Positive	0	1	

ly			0.211

Negative	10	7	

Positive	0	3	

V			1.000

Negative	9	9	

Positive	1	1	

Lymph node harvest, median (range)	15.0 (3 to 30)	16.5 (3 to 23)	0.853

^a^C-LAC = conventional laparoscopic colectomy; S-LAC = single-port laparoscopic colectomy. Data are expressed as median (range) or as raw numbers.

### Statistical analysis

All continuous variables are expressed as the median (range) and were compared using the Mann-Whitney *U *test. The *χ*^2 ^test and Fisher's exact test were used to compare discrete variables. Statistical calculations were performed with the help of the SPSS version 18.0 software program (SPSS, Chicago, IL, USA), and a *P *value < 0.05 was considered to indicate statistical significance.

## Results

Twenty patients (8 males and 12 females) were enrolled in this study, and they were distributed into two groups: S-LAC and C-LAC. All patients were matched as closely as possible in terms of their selection criteria. The data for both groups are shown in Table 3. There was no surgical mortality or reintervention within 30 days in either group. There were no significant differences in the lengths of the operations between the S-LAC group (median 192 minutes, range 156 to 231 min) and the C-LAC group (median 222 minutes, range 44 to 244 minutes). There also were no significant differences in the estimated blood loss between the S-LAC group (median 48.0 ml, range 0 to 110 ml) and the C-LAC group (median 51.5 ml, range 21 to 244 ml). Although there was one conversion to open surgery in the C-LAC group due to anatomical difficulties, there were no conversions in the S-LAC group. Regarding the length of the skin incision, that in the S-LAC group (median 3.0 cm, range 2.0 to 3.0 cm) was significantly shorter than that of the C-LAC group (median 5.0 cm, range 3.0 to 6.0 cm; *P *< 0.001). In terms of the hospital stay, the median stay of 8.0 days in the S-LAC group (range 6 to 13 days) was significantly shorter than the median of 10.5 days in the C-LAC group (range, 7 to 21 days; *P *= 0.023), as shown in Table 1. There were no surgical complications, including anastomotic leakage, surgical site infection, ileus, pneumonia, liver and renal dysfunction, or cardiovascular disease in either group (data not shown). With regard to the pathological findings, including the tumor differentiation, depth of the tumor, node metastasis, lymphatic invasion and vascular invasion, there were no significant differences between the groups. Moreover, the median number of lymph nodes extracted was also not significantly different between the S-LAC group (median 15.0, range 3 to 30) and the C-LAC group (median 16.5, range 3 to 23), as shown in Table 2.

**Table 3:** Preoperative parameters of patients^a^

	Laparoscopic colectomy for colon cancer		
**Demographics**	**S-LAC**	**C-LAC**	***P *value**

Number of Patients	10	10	

Age (years)	68.5 (61 to 81)	68.0 (33 to 84)	0.853

Sex			1.000

Male	4	4	

Female	6	6	

BMI (kg/m^2^)	22.5 (19.6 to 24.6)	21.9 (17.1 to 26.2)	0.353

ASA score			1.000

1	8	7	

2	2	3	

Prior abdominal surgery rate (%)	2 (20%)	3 (0%)	1.000

Type (Japanese Society for Cancer of the Colon and Rectum, 7th edition)			0.087

0	10	6	

1	0	3	

2	0	1	

Location			0.057

C (Cecum)	5	1	

A (Ascending colon)	4	9	

T (Transverse colon)	1	0	

^a^ASA = American Society of Anesthesiologists; BMI = body mass index; C-LAC = conventional laparoscopic colectomy; S-LAC = single-port laparoscopic colectomy. Data are expressed as median (range) or as raw numbers.

## Discussion

The use of single-port laparoscopic cholecystectomy has spread rapidly, and many procedures have already been performed throughout the world. On the other hand, single-port laparoscopic colon surgery for colon cancer has not yet been standardized. There are only a few reports of small sample size studies in the literature [6-14]. It has been suggested that single-port laparoscopic colectomy for colon cancer provides a better cosmetic outcome for patients than conventional laparoscopic surgery, with equivalent invasiveness between the procedures. However, there has been no adequate evidence regarding not only these issues but also the feasibility and safety of this operation. In this study, we compared various parameters between S-LAC and C-LAC to evaluate the feasibility and safety, as well as the outcomes, of single-port laparoscopic colectomy for colon cancer which required D2 lymph node dissection.

The apparent advantage of single-port laparoscopic colectomy is a better cosmetic outcome. Our data also reveal that the median length of the skin incision in the S-LAC group of 3.0 cm (range 2.0 to 3.0 cm) was significantly shorter than that of 5.0 cm in the C-LAC group (range 3.0 to 6.0 cm) (*P *< 0.001). To evaluate the invasiveness of the procedure, we compared the length of the operation, estimated blood loss and hospital stay. In our series, there were no significant differences between the S-LAC and C-LAC groups regarding the length of the operation or estimated blood loss. In terms of the hospital stay, the median of 8.0 days in the S-LAC group (range 6 to 13 days) was significantly shorter than the median of 10.5 days in the C-LAC group (range 7 to 21 days) (*P *= 0.023). Generally, the duration of the hospital stay has been used as one of the most important parameters of invasiveness. However, the hospital stay is defined not only by the patient's situation but also based on the characteristics of many Japanese patients who hope to stay for a long period in the hospital. Hence, the hospital stay is not necessarily a reliable parameter on which to objectively assess the invasiveness of such patients. However, these findings demonstrate that S-LAC is not more invasive than C-LAC or open colectomy.

The main disadvantage of this procedure is the difficulty in performing it, owing to the lack of instrument triangulation, clashing of the instruments outside the abdomen, a requirement for articulated instruments and the potential for pneumoperitoneum leaks. To resolve these problems, we primarily use the Gelport as the access port. In other words, the most important point for ensuring successful single-port laparoscopic colectomy is the selection of the access port to use. Initially, the multiple fascial puncture technique under a skin flap [15] was used for single-incision laparoscopic surgery, especially for cholecystectomy. However, the disadvantages of this technique are the weakness of the fascia due to the creation of multiple defects, as well as seroma formation. Therefore, several new access ports have already been developed. We usually use the Gelport, which has been used for hand-assisted laparoscopic surgery, as the access port for single-port laparoscopic colectomy. The benefit of using the Gelport is that several trocars can be inserted multiple times if necessary, and the trocars can be kept apart for as long as possible to maintain instrument triangulation and to prevent instrument clashing outside the abdomen. The most important issue affecting single-port laparoscopic colectomy is the much smaller space outside the abdomen than is present during conventional laparoscopic surgery. This difficult situation requires the use of articulated instruments. However, we did not need to use any articulated instruments when we used the Gelport as the access port. Moreover, the Gelport was able to maintain an airtight seal during the operation. Therefore, we concluded that our method using the Gelport has the potential to successfully address these limitations [16].

Our series of single-port laparoscopic colectomies for colon cancers (*n *= 10) had no conversions (Table 1) and no surgical complications, including anastomotic leakage, surgical site infection, ileus, pneumonia, cardiovascular disease and so on. These results revealed the feasibility and safety of single-port laparoscopic colectomy for colon cancer during the perioperative period.

In terms of the median number of extracted lymph nodes, there were no significant differences between the S-LAC group (median 15.0, range 3 to 30) and the C-LAC group (median 16.5, range 3 to 23) (*P *= 0.912), as shown in Table 2. These results demonstrate the feasibility regarding the short-term oncologic outcomeof single-port laparoscopic colectomy for colon cancer which requires D2 lymph node dissection.

This study is limited by its small sample size. However, it provides an initial comparison between S-LAC and C-LAC and can provide the foundation for large, randomized controlled studies.

## Conclusion

Our early experiences indicates that S-LAC for right-sided colon cancer is a feasible and safe procedure. Although there were no significant benefits regarding the perioperative and oncological results, S-LAC does provide a better cosmetic outcome. Before extending the indications of this procedure to advanced cases and those with rectal cancer, however, it will be necessary to evaluate this technique's perioperative and long-term oncological safety in a large, randomized controlled trial.

## Abbreviations

ASA: American Society of Anesthesiologists; BMI: Body mass index; C-LAC: Conventional laparoscopic colectomy; NOTES: Natural orifice transluminal endoscopic surgery; S-LAC: Single-port laparoscopic colectomy.

## Competing interests

The authors declare that they have no competing interests.

## Authors' contributions

HE participated in the treatment of these patients and the literature search and drafted the manuscript. MH helped to draft the manuscript. TH, YT, YK, MS, MT, TA, TU and TI participated in the treatment of these patients. HO participated in treatment planning for these patients and helped to draft the manuscript. All authors read and approved the final manuscript.
